# FRMD: fast robot motion diffusion via trajectory-level consistency distillation

**DOI:** 10.3389/frobt.2026.1751688

**Published:** 2026-03-25

**Authors:** Xirui Shi, Yi Hu, Jun Jin

**Affiliations:** 1 Department of Electrical and Computer Engineering, University of Alberta, Edmonton, AB, Canada; 2 Alberta Machine Intelligence Institute (Amii), Edmonton, AB, Canada

**Keywords:** consistency models, diffusion models, motion generation, movement primitives, robot learning, robotic manipulation

## Abstract

Foundation models for embodied artificial intelligence (Embodied AI) increasingly adopt diffusion modules as the action generation core of vision–language–action (VLA) policies, but the diffusion module’s iterative denoising imposes prohibitive inference latency for real-time deployment. We address this bottleneck in isolation by rethinking the *diffusion action generation module* itself. We present ***Fast Robot Motion Diffusion (FRMD)***, a fast robot motion diffusion framework that (i) operates in *trajectory-parameter* space by predicting movement-primitive coefficients in a low-dimensional manifold, and (ii) collapses multi-step sampling into a *single inference step* via *trajectory-level consistency distillation* over the probability-flow ordinary differential equation (ODE). Concretely, FRMD replaces stepwise action generation with a one-pass mapping from noise to full trajectories, followed by a fixed-cost basis expansion; this reduces policy latency from hundreds to tens of milliseconds without modifying upstream vision or language encoders. On standard robotic manipulation task benchmarks, FRMD attains 7 times faster than the vanilla diffusion policy and 10 times faster than the state-of-the-art MPD method, while matching the task success of multi-step diffusion policies. By targeting the diffusion component used throughout VLA systems, FRMD provides a plug-in, latency-optimized motion generator that preserves the advantages of diffusion and makes real-time embodied AI feasible.

## 1 Introduction

Robotic foundation models have recently emerged as a promising approach for embodied artificial intelligence, enabling robots to acquire generalizable manipulation skills through large-scale pre-training (Black et al., 2024; Driess et al., 2023). Among these models, vision-language-action (VLA) architectures have shown strong performance across diverse manipulation tasks, with diffusion-based policies becoming a common choice for action generation (Chi et al., 2023). Diffusion models are attractive in this setting because they can represent complex, multi-modal action distributions, which are essential for tasks that admit multiple valid behaviors, such as bimanual manipulation or tool use.

Despite these advantages, diffusion-based policies face a major limitation in robotic deployment: inference speed. Standard diffusion samplers require tens of denoising steps per action prediction (Chi et al., 2023; Song et al., 2020), leading to inference rates on the order of 1–2 Hz on typical robotic hardware. Such latency is incompatible with real-time control in dynamic settings, where robots must respond quickly to contact events, external disturbances, or changes in the environment. As a result, while vision and language components of robotic foundation models continue to scale, action generation remains a primary computational bottleneck.

In this work, we address this bottleneck by accelerating only the action-generation module of diffusion-based VLA policies. Rather than modifying the full perception or language pipeline, we focus on compressing the diffusion decoder itself, while reusing established observation encoders from prior work (Chi et al., 2023). This design choice allows our method to integrate directly with existing VLA architectures and serve as a drop-in replacement for standard diffusion action generators.

We propose ***Fast Robot Motion Diffusion (FRMD)***, a trajectory-level consistency distillation framework that converts a pre-trained multi-step diffusion policy into a few-step generator suitable for real-time control. FRMD builds on consistency models (Song et al., 2023) by enforcing self-consistency along the probability-flow ODE: any intermediate diffusion state is mapped to the same underlying action trajectory. In contrast to image-generation settings, FRMD is designed for robotic control, where actions must be temporally correlated, multi-modal at the trajectory level, and produced at control frequency. By learning a consistency function over action sequences, FRMD achieves 1–4 step generation with minimal loss in task performance. We evaluate FRMD on 12 manipulation tasks drawn from well recognized benchmarks–MetaWorld and ManiSkill (4 easy, 5 medium, 3 hard), using 100 demonstrations per benchmark and reporting both task success rate and wall-clock latency. FRMD attains an ***average policy latency of 17.2 ms*** across tasks while maintaining or improving task success relative to multi-step diffusion baselines. We also include a small architectural ablation (transformer, lightweight transformer, MLP) to illustrate compute/accuracy trade-offs. By isolating and accelerating the diffusion motion module, FRMD turns diffusion-based action generators into practical low-latency components for embodied foundation models, without requiring changes to upstream vision or language components.

Our contributions are summarized as follows.

- **Consistency distillation in structured motion parameter space.**

We propose Fast Robot Motion Diffusion (FRMD), a framework that applies consistency distillation to diffusion-based robotic policies using structured motion representations. Instead of distilling directly in raw action space, FRMD performs distillation in the parameter space of Probabilistic Dynamic Movement Primitives (ProDMPs), where temporal structure is explicitly encoded through basis functions.

- **Single-step trajectory generation with real-time latency.**

FRMD generates complete action trajectories in a single forward pass, achieving an average inference latency of 17.2 ms—approximately 10 × faster than multi-step diffusion-based teachers and 7 × faster than standard diffusion policies. Despite this acceleration, FRMD preserves task performance, matching or exceeding the success rates of the teacher model while significantly reducing non-smooth action transitions across 12 manipulation tasks.

- **Modular and encoder-agnostic integration.**

FRMD is designed as a drop-in replacement for the diffusion action decoder in vision-language-action pipelines. The consistency model and ProDMPs-based decoder operate independently of the perception and language encoders, enabling straightforward integration with existing or future VLA architectures without retraining the full system.

## 2 Related work

Our work is related to prior research on diffusion-based robot policies, consistency models for fast generative sampling, and structured motion representations such as movement primitives. The comparison between our methods and existed methods are shown in Table 1.

**TABLE 1:** Comparison of FRMD with prior paradigms.

Category	Limitations	FRMD (ours)
**Action-Space Diffusion** (Janner et al., 2022; Chi et al., 2023; Black et al., 2024)	High-dimensional action space (nk) ; 10–100 denoising steps; latency > 100 ms	Low-dimensional MP space (d≪nk) ; single-step inference; 17 ms runtime ( ∼7× speedup)
**Movement Primitives** (Ijspeert et al., 2013; Paraschos et al., 2013; Doe and Smith, 2017)	Deterministic or hand-tuned; limited expressiveness for complex distributions	Learns stochastic diffusion over MPs weights; scalable to multi-modal behaviors
**Consistency Models** (Song et al., 2023; Kim et al., 2023; Dai et al., 2024)	Limited to vision/language domains; no robotic control validation	Trajectory-level consistency distillation for robotics
**Trajectory-Parameterized Diffusion** (Carvalho et al., 2024; Scheikl et al., 2024)	Multi-step denoising remains; not real-time feasible	One-step consistency mapping; achieves real-time control with diffusion ( ∼10× speedup)

FRMD, combines trajectory-space diffusion with consistency distillation to achieve real-time inference while maintaining task success.

### 2.1 Diffusion models for robot learning

Diffusion models have recently become a common mechanism for action generation in robot learning, adapting denoising diffusion techniques from computer vision (Ho et al., 2020) to sequential decision-making. Planning-as-generation methods and diffusion policies generate action sequences through iterative denoising conditioned on observations and, in some cases, action history (Janner et al., 2022; Chi et al., 2023). These approaches demonstrate strong performance in multi-modal manipulation tasks and have been adopted in large-scale vision-language-action models such as $\pi_{0}$ (Black et al., 2024).

A key limitation of most diffusion-based robot policies is that they operate directly in high-dimensional action space and require multiple denoising steps for each prediction. Even with lightweight perception backbones, this results in inference latencies that exceed real-time control budgets. In addition, raw action-space parameterization does not explicitly encode trajectory-level structure, making it difficult to collapse iterative sampling into a single forward pass.

### 2.2 Consistency models for fast diffusion

Consistency models were introduced as a means of accelerating diffusion sampling while preserving generation quality (Song et al., 2023). By enforcing self-consistency along the probability-flow ODE, these models enable noisy inputs at arbitrary diffusion times to be mapped directly to clean outputs, eliminating the need for iterative denoising. Subsequent work has demonstrated substantial speedups in image and text-to-image generation, including latent consistency models and motion-related extensions (Kim et al., 2023; Dai et al., 2024; Chen et al., 2024).

Most existing consistency models are developed for image or language domains, where the generation space is either pixel-based or low-dimensional latent representations. Applying consistency distillation to robotic control presents additional challenges, as robot actions are temporally correlated, high dimensional, and must be generated at control frequency. As a result, the use of consistency models for fast action generation in robotics remains relatively unexplored.

### 2.3 Movement primitives in robotics

Movement primitives (MPs) provide structured representations for generating smooth and temporally coherent robot motion. Classical Dynamic Movement Primitives (DMPs) (Ijspeert et al., 2013; Saveriano et al., 2023) encode motion through dynamical systems with nonlinear forcing terms, enabling invariance to timing and goal position. Probabilistic Movement Primitives (ProMPs) (Paraschos et al., 2013) extend this formulation by modeling trajectories as distributions over basis-function weights, capturing correlations across degrees of freedom. ProDMPs (Doe and Smith, 2017) further improve efficiency by precomputing basis expansions, allowing deterministic and fast trajectory decoding.

Movement primitives provide a compact and structured representation of trajectories. However, they are typically used in low-dimensional settings and are not commonly applied to high-dimensional stochastic policy learning in recent robot learning pipelines. Diffusion-based policies, on the other hand, can model complex observation spaces and multi-modal action distributions, but require iterative sampling and therefore incur higher computational cost, while offering less explicit trajectory structure.

### 2.4 Diffusion with trajectory parameterization

Recent work has explored combining diffusion models with parameterized trajectory representations, such as movement-primitive weight spaces, instead of modeling raw action sequences (Carvalho et al., 2024; Scheikl et al., 2024). These approaches leverage the structure of MPs to generate smoother and more stable trajectories, particularly in manipulation and deformable-object tasks. However, they continue to rely on multi-step denoising during inference, which remains a major source of latency.

Existing trajectory-parameterized diffusion methods primarily focus on motion quality and generalization, and do not address the computational cost of iterative sampling. As a result, despite improved trajectory structure, these approaches are still unsuitable for real-time control scenarios that require fast action generation.

## 3 Methodology

This section presents Fast Robot Motion Diffusion (FRMD), our approach for efficient generation of smooth robot trajectories. Table 2 summarizes the key notation used throughout this section.

**TABLE 2:** Notation and symbols used throughout this paper.

Symbol	Definition	Symbol	Definition
*Problem Formulation*		*Movement Primitives (cont.)*	
n	Number of time steps in trajectory	$c_{1},c_{2}$	Coefficients from boundary conditions
k	Action dimensionality (robot DoF)	Φ(t)	Vector of basis functions, $\in R^{d}$
τ	Action trajectory, $\in R^{n\times k}$	$\phi_{i}\left(t\right)$	Individual basis function
$a_{i}$	Action at time step i , $\in R^{k}$	G	ProDMPs decoder function
o	Observation (images + proprioception)		
O	Observation space	*Diffusion and Consistency Models*	
A	Action space, $R^{n\times k}$	s	Diffusion time, s∈[0,S]
$\pi_{\theta }$	Robot policy	S	Maximum diffusion time
D	Dataset of demonstrations	σ(s)	Noise schedule at diffusion time s
M	Number of demonstrations	$\dot{\sigma }\left(s\right)$	Time derivative of noise schedule
		$\sigma_{\max}$	Maximum noise level
*Movement Primitives*		$\sigma_{\text{data}}$	Estimated data standard deviation
d	Dimension of MP weight space	ϵ	Gaussian noise, ∼N(0,I)
w	MP weight vector, $\in R^{kd}$	$x_{s}$	Data sample at diffusion time s
W	Weight matrix, $\in R^{k\times d}$	$p_{s}\left(x\right)$	Distribution at diffusion time s
t	Continuous trajectory time	$\nabla_{x}\log p_{s}\left(x\right)$	Score function
T	Time horizon	ϵ	Small constant (avoid instability)
y(t)	Position at time t (single DoF)		
$\dot{y}\left(t\right)$	Velocity at time t (single DoF)	*Network Architecture*	
$y_{0},{\dot{y}}_{0}$	Initial position and velocity	θ	Student (online) network parameters
$\psi_{1}\left(t\right),\psi_{2}\left(t\right)$	Homogeneous basis functions	$\theta^{-}$	Target (EMA) network parameters
*Network Architecture (cont.)*		*Training Parameters*	
$E_{\theta }$	Encoder network	μ	EMA decay rate (typically 0.95)
$F_{\theta }$	Complete model (encoder + decoder)	η	Learning rate
$f_{\theta }$	Consistency function (student)	K	Number of teacher steps
$f_{\theta^{-}}$	Target consistency function (EMA)	N	Total discretization steps
$F_{\text{teacher}}$	Pre-trained teacher model	$\sigma_{i}$	Discretized noise level at step i
$c_{\text{skip}}\left(s\right)$	Skip connection coefficient	n	Sampled time step index
$c_{\text{out}}\left(s\right)$	Output scaling coefficient	Ψ	ODE solver for teacher
		$L_{\text{teacher}}$	Teacher training loss
		$L_{\text{CD}}$	Consistency distillation loss
		d(⋅,⋅)	Distance metric (e.g., $L_{2}$ )
		$\lambda \left(\sigma_{n}\right)$	Weighting function (consistency)
		ω(s)	Weighting function (score matching)

**FIGURE 1:**
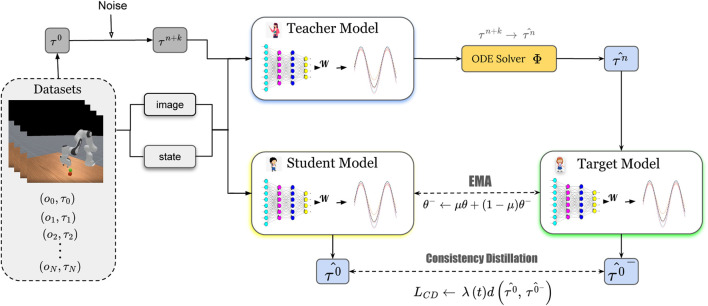
Overview of FRMD Training Framework. Given observations $o_{i}$ , raw action sequence $\tau^{0}$ and initial state $\left(y_{0},\dot{y_{0}}\right)$ from the robot datasets, we first perform a forward diffusion to introduce noise over n+k steps. The resulting noisy sequence $\tau^{n+k}$ is then fed into both the student model and the teacher model to predict the action sequence $\tau^{0}$ and $\tau^{n}$ . The target model uses the teacher network’s k -step estimation results to predict the action sequence. The student model, trained via consistency distillation and its weights are updated through an Exponential Moving Average (EMA).

We begin with the problem formulation, introduce the foundational concepts of Movement Primitives and Consistency Models, and describe how FRMD synthesizes these techniques. Figure 1 provides an overview of the proposed framework.

### 3.1 Problem formulation

We aim to learn a robot policy capable of generating smooth, structured action sequences efficiently from sensory observations.

**Action Trajectory:** A robot motion trajectory is defined as an ordered sequence of actions over a finite time horizon as formalized in Equation 1:

$$\tau =\left[a_{0},a_{1},\ldots ,a_{n-1}\right]\in R^{n\times k}$$ (1)

where n denotes the number of discrete time steps, $a_{i}\in R^{k}$ represents the action at time step i , and k is the action dimensionality determined by the robot’s control mode and degrees of freedom (DoFs). For instance, when controlling a 7-DoF manipulator with joint position commands, k=7 .

**Policy Learning:** Given a dataset of expert demonstrations $D={\left\{\left(o_{j},\tau_{j}\right)\right\}}_{j=1}^{M}$ , we seek to learn a policy $\pi_{\theta }$ that maps observations to action sequences as formalized in Equation 2:

$$\pi_{\theta }:O\to A$$ (2)

where O denotes the observation space and $A=R^{n\times k}$ is the action space. Observations o∈O typically comprise RGB images from onboard cameras and proprioceptive information such as joint positions and velocities. Diffusion models typically require multiple iterative denoising steps, which incurs substantial inference costs, rendering them impractical for real-time robotic control.

### 3.2 Preliminaries

#### 3.2.1 Movement primitives

We briefly review Probabilistic Dynamic Movement Primitives (ProDMPs), which we use as a structured parameterization of action trajectories.

**Probabilistic Dynamic Movement Primitives.** ProDMPs (Ijspeert et al., 2013; Saveriano et al., 2023) represent a trajectory as a linear combination of predefined basis functions. For a single degree of freedom, the position y(t) at continuous time t∈[0,T] is given by:

$$y\left(t\right)=c_{1}\left(y_{0},{\dot{y}}_{0}\right)\;\psi_{1}\left(t\right)+c_{2}\left(y_{0},{\dot{y}}_{0}\right)\;\psi_{2}\left(t\right)+\Phi {\left(t\right)}^{⊤}w,$$ (3)

where.

-
$\psi_{1}\left(t\right)$ and $\psi_{2}\left(t\right)$ are homogeneous solutions of the underlying second-order linear dynamical system.
-
$c_{1}\left(\cdot \right)$ and $c_{2}\left(\cdot \right)$ are coefficients determined by the initial position $y_{0}=y\left(0\right)$ and velocity ${\dot{y}}_{0}=\dot{y}\left(0\right)$ .
-
$\Phi \left(t\right)={\left[\phi_{1}\left(t\right),\ldots ,\phi_{d}\left(t\right)\right]}^{⊤}$ denotes a set of predefined basis functions, typically Gaussian radial basis functions distributed over time.
-
$w\in R^{d}$ is a weight vector that parameterizes the trajectory.

The corresponding velocity trajectory is obtained by differentiation:

$$\dot{y}\left(t\right)=c_{1}\left(y_{0},{\dot{y}}_{0}\right)\;{\dot{\psi }}_{1}\left(t\right)+c_{2}\left(y_{0},{\dot{y}}_{0}\right)\;{\dot{\psi }}_{2}\left(t\right)+\dot{\Phi }{\left(t\right)}^{⊤}w.$$ (4)

For a system with k degrees of freedom, ProDMPs are applied independently to each dimension, resulting in a weight matrix $W\in R^{k\times d}$ . For convenience, we vectorize this matrix as $w\in R^{kd}$ in the following sections.

**Decoder Mapping.** The mapping from ProDMP weights to a discrete action trajectory is defined as:

$$\tau =G\left(w;y_{0},{\dot{y}}_{0}\right),$$ (5)

where G denotes the deterministic ProDMP decoder that evaluates Equations 3, 4 across time steps and degrees of freedom to produce the full action sequence $\tau \in R^{n\times k}$ .

#### 3.2.2 Consistency models

Consistency models (Song et al., 2023) are an alternative to standard diffusion models that aim to reduce inference cost by learning direct mappings from noisy inputs to clean data. Instead of generating samples through iterative denoising, a consistency model predicts the final output in a single forward pass conditioned on the noise level.

**Probability Flow ODE.** Diffusion models can be formulated as solving a continuous-time ordinary differential equation (ODE), known as the probability flow ODE (PF-ODE). Given a data sample $x_{0}∼p_{\text{data}}$ , the forward noising process is defined as Equation 6:

$$x_{s}=x_{0}+\sigma \left(s\right)\epsilon ,\;\epsilon ∼N\left(0,I\right),$$ (6)

where s∈[0,S] denotes diffusion time (distinct from trajectory time t ), and σ(s) is a monotonically increasing noise schedule with σ(0)=0 and $\sigma \left(S\right)=\sigma_{\max}$ . The corresponding reverse process is governed by the PF-ODE as formalized in Equation 7:

$$\frac{dx}{ds}=-\dot{\sigma }\left(s\right)\sigma \left(s\right)\nabla_{x}\log p_{s}\left(x\right),$$ (7)

where $\nabla_{x}\log p_{s}\left(x\right)$ is the score function of the perturbed data distribution. Numerically integrating this ODE from s=S to s=0 recovers a clean sample from an initial Gaussian noise input.

**Consistency Function.** A consistency model parameterizes a function $f_{\theta }\left(x_{s},s\right)$ that predicts the corresponding clean sample $x_{0}$ from any noisy state $x_{s}$ along the PF-ODE trajectory. The defining property is self-consistency as showed in Equation 8: predictions from different diffusion times along the same trajectory should coincide,

$$f_{\theta }\left(x_{s},s\right)=f_{\theta }\left(x_{s^{\prime }},s^{\prime }\right),\;\forall s,s^{\prime }\in \left[\epsilon ,S\right],$$ (8)

with the boundary condition as formalized in Equation 9:

$$f_{\theta }\left(x_{0},0\right)=x_{0},$$ (9)

ensuring that clean inputs are fixed points of the mapping.

**Consistency Distillation.** The consistency function is trained by distilling a pre-trained diffusion model using a student–teacher setup. The student network $f_{\theta }$ learns to predict clean data directly from noisy inputs, while a target network $f_{\theta^{-}}$ provides stable training targets. The target network shares the same architecture as the student and is updated as an exponential moving average (EMA) of the student parameters as formalized in Equation 10:

$$\theta_{t+1}^{-}=\mu \theta_{t}^{-}+\left(1-\mu \right)\theta_{t},$$ (10)

where $\mu \in \left.\left[0,1\right.\right)$ is the EMA decay rate.

Training samples are generated by first adding noise to clean data and then applying the teacher diffusion model for a small number of steps to obtain intermediate denoised states. The student prediction from a noisy input is encouraged to match the target network’s prediction from the teacher-generated intermediate state. This objective enforces consistency across diffusion times and enables one-step inference at test time.

The resulting consistency model replaces iterative denoising with a single forward evaluation, serving as a fast alternative to standard diffusion sampling.

### 3.3 Fast robot motion diffusion (FRMD)

FRMD combines structured motion representations with consistency-based distillation to enable fast trajectory generation for robot control. The method follows a two-stage training procedure: we first train a diffusion model in the movement-primitive weight space, and then distill it into a consistency model that performs single-step inference.

#### 3.3.1 Teacher model: Diffusion in movement primitive space

The teacher model performs diffusion in the movement-primitive (MP) weight space rather than directly in action space. Instead of denoising high-dimensional action sequences $\tau \in R^{n\times k}$ , the model operates on compact MP weight vectors $w\in R^{kd}$ , which are subsequently decoded into smooth trajectories using the ProDMP formulation.

The teacher model $F_{\theta }$ consists of a learned encoder and a deterministic decoder. The encoder network $E_{\theta }:R^{n\times k}\times O\times R_{+}\to R^{kd}$ takes as input a noisy trajectory $\tilde{\tau }$ , the observation o , and the diffusion time s , and predicts denoised MP weights. These weights are then mapped to a full action trajectory using the ProDMP decoder G defined in Equation 5:

$$F_{\theta }\left(\tilde{\tau },o,s\right)=G\left(E_{\theta }\left(\tilde{\tau },o,s\right);y_{0},{\dot{y}}_{0}\right).$$ (11)

The movement-primitive hyperparameters used by the ProDMPs decoder, such as the number and temporal placement of basis functions, are specified offline following standard ProDMP formulations and are kept fixed throughout training and inference. Trajectory generation follows the probability flow ODE adapted to the conditional setting:

$$\frac{d\tau }{ds}=-\dot{\sigma }\left(s\right)\sigma \left(s\right)\nabla_{\tau }\log p_{s}\left(\tau |o\right),$$ (12)

where $p_{s}\left(\tau |o\right)$ denotes the distribution of trajectories conditioned on the observation at diffusion time s . Using Tweedie’s formula (Efron, 2011), the score function is approximated as formalized in Equation 13.

$$\nabla_{\tau }\log p_{s}\left(\tau |o\right)\approx \frac{F_{\theta }\left(\tau ,o,s\right)-\tau }{\sigma {\left(s\right)}^{2}}.$$ (13)

The teacher model is trained using denoising score matching. Given a clean trajectory $\tau^{0}$ and boundary conditions $\left(y_{0},{\dot{y}}_{0}\right)$ , a diffusion time s is sampled and Gaussian noise is added to obtain $\tilde{\tau }=\tau^{0}+\sigma \left(s\right)\epsilon$ , with ϵ∼N(0,I) . The training objective is Equation 14.

$$L_{\text{teacher}}\left(\theta \right)=E_{s,\tau^{0},\epsilon }\left[\omega \left(s\right){\left\|F_{\theta }\left(\tilde{\tau },o,s\right)-\tau^{0}\right\|}_{2}^{2}\right],$$ (14)

where $\omega \left(s\right)=1/\sigma {\left(s\right)}^{2}$ follows the standard score-matching weighting.

At inference time, the teacher generates trajectories by sampling an initial noise trajectory $\tau_{S}∼N\left(0,\sigma_{\max}^{2}I\right)$ and numerically integrating the ODE in Equation 12 from s=S to s=0 using a high-order solver such as DPM-Solver (Lu et al., 2022), yielding the final trajectory $\tau_{0}$ .

#### 3.3.2 Student model: One-step consistency distillation

The teacher diffusion model described above produces high-quality trajectories but requires multiple ODE integration steps at inference time. To remove this iterative sampling cost, we distill the teacher into a student consistency model that generates full trajectories in a single forward pass.

**Student–Target Architecture.** We adopt a student–target training setup following prior work on consistency models. The student network $f_{\theta }$ is trained by gradient descent to predict clean trajectories from noisy inputs at arbitrary diffusion times. A second network, $f_{\theta^{-}}$ , shares the same architecture but serves as a slowly updated target. The target network is not directly optimized; instead, its parameters are updated as an exponential moving average (EMA) of the student parameters. Both networks are initialized from the pre-trained teacher model:

$$\theta^{\left(0\right)}=\theta^{-\left(0\right)}=\theta_{\text{teacher}}.$$

This initialization ensures that the student begins training from a trajectory generator that already produces valid motions.

**Consistency Function Parameterization.** Both the student and target networks are parameterized using the standard consistency-model formulation with skip connections as formalized in Equation 15.

$$f_{\theta }\left(\tau ,o,s\right)=c_{\text{skip}}\left(s\right)\;\tau +c_{\text{out}}\left(s\right)\;F_{\theta }\left(\tau ,o,s\right),$$ (15)

where $F_{\theta }$ denotes the same encoder–decoder architecture used in the teacher model Equation 11. The target network $f_{\theta^{-}}$ uses the same functional form with parameters $\theta^{-}$ .

The coefficients are formalized in Equation 16.

$$c_{\text{skip}}\left(s\right)=\frac{\sigma_{\text{data}}^{2}}{\sigma {\left(s\right)}^{2}+\sigma_{\text{data}}^{2}},\;c_{\text{out}}\left(s\right)=\frac{\sigma \left(s\right)\sigma_{\text{data}}}{\sqrt{\sigma {\left(s\right)}^{2}+\sigma_{\text{data}}^{2}}},$$ (16)

are chosen so that the boundary condition

$$f_{\theta }\left(\tau^{0},0\right)=\tau^{0}$$

is satisfied. This guarantees that clean trajectories are fixed points of the mapping and ensures numerical stability near s=0 .

**Trajectory-Level Consistency Distillation.** Training samples are constructed by first corrupting a clean trajectory $\tau^{0}$ with Gaussian noise at diffusion index n+K :

$$\tau^{n+K}=\tau^{0}+\sigma_{n+K}\epsilon ,\;\epsilon ∼N\left(0,I\right).$$

Starting from this noisy input, the teacher diffusion model is applied for K probability-flow ODE steps to obtain an intermediate trajectory as formalized in Equation 17:

$${\hat{\tau }}^{n}=\Psi \left(\tau^{n+K},\sigma_{n+K},\sigma_{n};F_{\text{teacher}},o\right),$$ (17)

where Ψ denotes a numerical ODE solver.

The student network predicts a clean trajectory directly from the highly noisy input,

$${\hat{\tau }}^{0,\text{student}}=f_{\theta }\left(\tau^{n+K},o,\sigma_{n+K}\right),$$

while the target network predicts from the teacher-generated intermediate state,

$${\hat{\tau }}^{0,\text{target}}=f_{\theta^{-}}\left({\hat{\tau }}^{n},o,\sigma_{n}\right).$$

Enforcing agreement between these predictions encourages the model to produce identical outputs for different points along the same probability-flow ODE trajectory.

**Training Objective.** The student is trained by minimizing the consistency distillation loss as formalized in Equation 18:

$$L_{\text{CD}}\left(\theta ,\theta^{-}\right)=E_{n,\tau^{0},\epsilon }\left[\lambda \left(\sigma_{n}\right){\left\|f_{\theta }\left(\tau^{n+K},o,\sigma_{n+K}\right)-f_{\theta^{-}}\left({\hat{\tau }}^{n},o,\sigma_{n}\right)\right\|}_{2}^{2}\right],$$ (18)

where we use $\lambda \left(\sigma_{n}\right)=1$ in all experiments. After each gradient update of the student parameters, the target parameters are updated via EMA as formalized in Equation 19:

$$\theta^{-}\leftarrow \text{stopgrad}\left(\mu \theta^{-}+\left(1-\mu \right)\theta \right),$$ (19)

with decay rate μ . To summarize, we propose Student Model Consistency Distillation as detailed in Algorithm 1.

**Inference.** At test time, only the student network $f_{\theta }$ is retained. A trajectory is generated by sampling noise $\tau_{S}∼N\left(0,\sigma_{\max}^{2}I\right)$ and applying a single forward pass:

$$\tau_{0}=f_{\theta }\left(\tau_{S},o,\sigma_{\max}\right).$$

The target network is discarded after training.

Algorithm 1 Consistency Distillation for FRMD Student Model.

1: **Input:** Dataset D , pre-trained
   teacher $F_{\text{teacher}}$ , learning rate η , EMA rate μ , ODE solver Ψ , number of teacher steps K , noise schedule ${\left\{\sigma_{i}\right\}}_{i=0}^{N}$

2: **Initialize:** Student parameters $\theta \leftarrow \theta_{\text{teacher}}$ , target parameters $\theta^{-}\leftarrow \theta$

3: **repeat**
4: Sample $\left(o,\tau^{0}\right)∼D$

5: Sample n∼U{1,2,…,N−K}

6: Sample ϵ∼N(0,I)

7: Inject noise: $\tau^{n+K}\leftarrow \tau^{0}+\sigma_{n+K}\epsilon$

8://Teacher provides intermediate target
9: Denoise with teacher: ${\hat{\tau }}^{n}\leftarrow \Psi \left(\tau^{n+K},\sigma_{n+K},\sigma_{n};F_{\text{teacher}},o\right)$

10://Compute consistency loss
11: $L_{\text{CD}}\leftarrow \lambda \left(\sigma_{n}\right)\;d\left(f_{\theta }\left(\tau^{n+K},o,\sigma_{n+K}\right),f_{\theta^{-}}\left({\hat{\tau }}^{n},o,\sigma_{n}\right)\right)$

12://Update networks
13: $\theta \leftarrow \theta -\eta \nabla_{\theta }L_{\text{CD}}$

14: $\theta^{-}\leftarrow \text{stopgrad}\left(\mu \theta^{-}+\left(1-\mu \right)\theta \right)$

15: **until** convergence
16: **Return:** Student model $f_{\theta }$

## 4 Experiments and evaluation

We conduct experiments to evaluate Fast Robot Motion Diffusion (FRMD) across multiple manipulation tasks. This section is organized as follows: we first describe the experimental setup (Section 4.1), then present the main results comparing FRMD against state-of-the-art baselines (Section 4.2), followed by ablation studies analyzing architectural choices (Section 4.3), and finally discuss limitations and future directions (Section 4.4).

Our evaluation aims to answer four key questions: **Q1:** How does FRMD compare against state-of-the-art methods in terms of success rate and computational efficiency? **Q2:** Does FRMD achieve one-step inference without sacrificing motion quality compared to multi-step baselines? **Q3:** Can FRMD generate smooth and temporally consistent trajectories across diverse manipulation tasks of varying complexity? **Q4:** How do different backbone network architectures affect FRMD’s performance?

### 4.1 Experimental setup

#### 4.1.1 Environments and tasks

We evaluate FRMD on 12 manipulation tasks from two widely-used robotic benchmarks: MetaWorld (Yu et al., 2020) and ManiSkill (Mu et al., 2021). Following Lu et al. (2024), we categorize these tasks into three difficulty levels based on the complexity of required manipulation skills.

- **Easy (4 tasks):** Simple pick-and-place operations requiring basic grasping and positioning (e.g., PickCube-v1, Reach-v1).
- **Medium (5 tasks):** Tasks requiring coordinated manipulation and moderate precision (e.g., PushT-v1, Door-v1).
- **Hard (3 tasks):** Complex dexterous manipulation requiring fine-grained control and multi-stage execution (e.g., PegInsertion-v1, PlugCharger-v1).

#### 4.1.2 Dataset collection and preprocessing

**Data Collection.** For each task, we collect 100 expert demonstrations using pre-trained policies for MetaWorld environments and trajectory replay for ManiSkill environments. Each demonstration $D_{j}={\left\{\left(o_{t}^{\left(j\right)},a_{t}^{\left(j\right)}\right)\right\}}_{t=0}^{T_{j}}$ consists of observation-action pairs recorded over a complete task execution, where $T_{j}$ denotes the episode length for demonstration j .

**Data Preprocessing.** We segment each demonstration into overlapping training samples using a sliding window approach. Specifically, we predict action sequences of length n=12 (action horizon) conditioned on the previous m=3 visual observations (observation history). From each demonstration, we extract samples $\left(o_{t-m+1:t},\tau_{t:t+n-1}\right)$ where.

-
$o_{t-m+1:t}=\left[o_{t-m+1},\ldots ,o_{t}\right]\in R^{m\times H\times W\times C}$ represents the observation history (RGB images with size of width (W) and height (H), and C channels.)
-
$\tau_{t:t+n-1}=\left[a_{t},\ldots ,a_{t+n-1}\right]\in R^{n\times k}$ is the target action sequence

This preprocessing yields the complete training dataset $D={\left\{\left(o^{\left(i\right)},\tau^{\left(i\right)}\right)\right\}}_{i=1}^{M}$ used for all methods.

#### 4.1.3 Baseline methods

We compare FRMD against two state-of-the-art diffusion-based robot learning methods.

- **Diffusion Policy (DP)** (Chi et al., 2023): A multi-step diffusion model that directly generates action sequences $\tau \in R^{n\times k}$ in the original action space without dimensional reduction.
- **Movement Primitives Diffusion (MPD)** (Scheikl et al., 2024): A state-of-the-art (SOTA) diffusion model that operates in the low-dimensional movement primitive weight space $w\in R^{kd}$ rather than directly in action space. MPD serves as the teacher model for our consistency distillation framework.

Our goal is to demonstrate that FRMD achieves significantly faster inference than both baselines while maintaining or exceeding the motion quality and task success rate of MPD.

#### 4.1.4 Evaluation metrics

We assess model performance using three complementary metrics:

**Success Rate (SR).** We evaluate each method by executing 10 episodes with randomized initial conditions every 5,000 training steps. An episode is considered successful if the task-specific success criterion is satisfied (e.g., object placed within target region, peg fully inserted). We report the average success rate across all evaluation episodes throughout training until convergence. Higher success rates indicate better task performance.

**Inference Time.** We measure the average wall-clock time required to generate one action sequence (12 steps) during deployment. This includes all computation from receiving observations to outputting actions, excluding environment simulation time. To account for variability, we run each experiment with three random seeds (0, 1, 2) and report mean and standard deviation. Lower inference times indicate greater computational efficiency.

**Motion Smoothness.** To quantify trajectory quality, we adopt a geometric approach (Guillén Ruiz et al., 2020) that measures trajectory smoothness via curvature analysis. For a given end-effector trajectory, we compute the discrete curvature $\kappa_{i}$ at each waypoint i . Following standard practice in robotics, we classify a transition as non-smooth if $\kappa_{i}>\kappa_{\max}=1.0$ rad/m, where $\kappa_{\max}$ represents the maximum curvature achievable without violating joint velocity constraints. We report the total number of non-smooth transitions $N_{\text{non}-\text{smooth}}=|\left\{i:\kappa_{i}>\kappa_{\max}\right\}|$ in the generated trajectory. Lower values indicate smoother, more natural motions.

#### 4.1.5 Implementation details

**Observation Encoder.** RGB observations are encoded using a modified ResNet18 (He et al., 2016) that outputs 128-dimensional feature embeddings. Following prior work (Chi et al., 2023), we replace the global average pooling layer with spatial softmax pooling to retain spatial information and substitute Batch Normalization with Group Normalization to improve training stability under small batch sizes. The observation encoder is trained jointly with the policy network for all methods.

**Policy Architectures.** For Diffusion Policy (DP), we use a convolutional backbone with three layers of channel sizes [256, 512, 1024]. For Movement Primitive Diffusion (MPD) and FRMD, we adopt a transformer-based architecture similar to Dasari et al. (2024), consisting of six transformer layers with four attention heads per layer, a hidden dimension of 256, and a dropout rate of 0.3. This architecture is used to model temporal dependencies in trajectory generation.

**Movement Primitive Decoder.** For MPD and FRMD, trajectories are parameterized using Probabilistic Dynamic Movement Primitives (ProDMPs). We use d=20 Gaussian radial basis functions per degree of freedom, uniformly distributed over the trajectory horizon.

**Training Setup.** All models are trained using the AdamW optimizer (Loshchilov and Hutter, 2017) with a batch size of 128, an initial learning rate of $1\times 10^{-4}$ , and weight decay $1\times 10^{-6}$ . Training is performed for 30,000 steps with a cosine learning-rate schedule and 500 warmup steps. For FRMD, the exponential moving average (EMA) decay rate of the target network is set to μ=0.95 , and the teacher diffusion model performs K=2 denoising steps during consistency distillation.

All methods are implemented in PyTorch and trained on a single NVIDIA RTX 4090 GPU. Unless otherwise specified, the same training and evaluation settings are used across all models.

### 4.2 Main results

#### 4.2.1 Overall performance comparison

Table 3 presents the comprehensive comparison of success rates and inference times across all 12 tasks. FRMD achieves the highest overall success rate of 64.8%, outperforming MPD (64.1%) and substantially exceeding DP (50.1%). Remarkably, FRMD accomplishes this with an average inference time of only 17.2 ms, representing a 10 × speedup over MPD (168.6 ms) and a 7 × speedup over DP (119.7 ms).

**TABLE 3:** Success rate and inference time comparison across all 12 tasks.

Method	Easy (4)	Medium (5)	Hard (3)	Overall
**Success Rate (%)**				
DP	**99.3** ± 0.1	41.0 ± 3.2	10.1 ± 1.4	50.1
MPD	98.9 ± 0.3	64.8 ± 2.6	28.6 ± 2.9	64.1
FRMD (Ours)	99.2 ± 0.1	**66.3** ± 1.2	**29.0** ± 2.3	**64.8**
**Inference Time (ms)**				
DP	119.8 ± 1.4	121.3 ± 2.3	118.2 ± 3.6	119.7
MPD	162.7 ± 3.5	173.2 ± 3.6	169.9 ± 1.2	168.6
FRMD (Ours)	**15.2** ± 0.8	**18.6** ± 3.4	**17.9** ± 1.1	**17.2**

Results are averaged over three random seeds with standard deviations shown. The best results in each category are highlighted in **bold**, and second-best results are underlined.

##### 4.2.1.1 Analysis by task difficulty

*Easy Tasks.* On tasks with low manipulation complexity, all three methods achieve near-saturated performance, with success rates exceeding 98%. Diffusion Policy (DP) attains the highest average success rate at 99.3%, followed closely by FRMD at 99.2% and MPD at 98.9%. The small performance differences in this regime indicate that task success is largely insensitive to the choice of action-generation method when the required motion patterns are simple. Despite comparable success rates, FRMD operates at substantially lower inference latency (15.2 ms) than DP (119.8 ms), reflecting the computational advantage of single-step generation even when task difficulty is low.

*Medium Tasks.* As task difficulty increases, clearer differences emerge between methods. FRMD achieves a success rate of 66.3%, outperforming MPD (64.8%) and DP (41.0%). The gap between FRMD and DP widens substantially in this regime, while the difference between FRMD and its teacher MPD remains relatively small. This trend suggests that structured trajectory representations play a more important role as tasks require longer-horizon coordination or more precise contact handling. Notably, FRMD matches or slightly exceeds the performance of the multi-step teacher while maintaining significantly lower inference cost.

*Hard Tasks.* Performance differences are most pronounced on the hardest manipulation tasks. FRMD achieves a success rate of 29.0%, compared to 28.6% for MPD and 10.1% for DP. While absolute success rates are lower for all methods, FRMD consistently outperforms both baselines in this setting. In contrast to DP, whose performance degrades sharply with increasing task complexity, FRMD maintains stable inference latency (17.9 ms) across task categories, indicating that its single-step generation procedure scales consistently as task demands increase.

#### 4.2.2 Computational efficiency

The inference-time measurements in Table 3 report the latency of action generation for all methods across task difficulty levels. FRMD exhibits consistently low inference latency across all task categories, with an average of 15.2 ms on easy tasks, 18.6 ms on medium tasks, and 17.9 ms on hard tasks, resulting in an overall mean latency of 17.2 ms.

All methods—FRMD, MPD, and Diffusion Policy (DP)—use the same RGB observation format and the same observation encoder architecture. Consequently, the reported latency differences reflect only the cost of the action-generation module and are not influenced by perception or preprocessing.

Compared to Movement Primitive Diffusion (MPD), FRMD achieves an average speedup of 9.8 × , with speedup factors ranging from 9.1 × to 10.7 × depending on task difficulty. Relative to Diffusion Policy, FRMD provides an average speedup of 7.0×. These results indicate that replacing multi-step diffusion sampling with a single-step consistency model substantially reduces inference-time computation in the action-generation stage.

#### 4.2.3 Learning efficiency analysis

**FIGURE 2:**
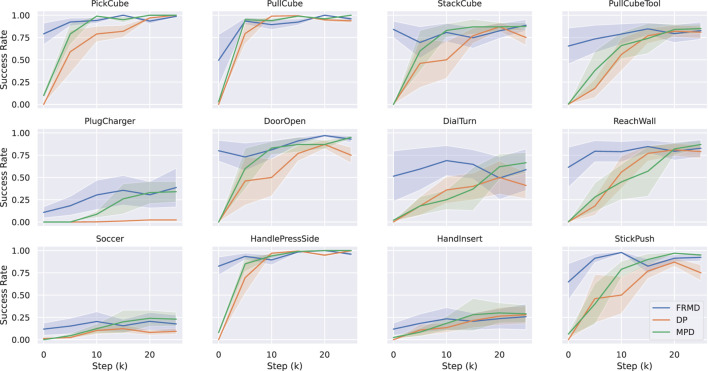
Learning curves across 12 manipulation tasks. We compare FRMD (ours), Diffusion Policy (DP), and Movement Primitives Diffusion (MPD) across tasks from MetaWorld and ManiSkill benchmarks. Success rates are computed by evaluating 10 episodes every 5,000 training steps until convergence. Solid lines represent mean success rates, and shaded regions indicate standard deviations across three random seeds. FRMD consistently achieves higher or comparable success rates while maintaining 10 × faster inference than MPD and 7 × faster than DP (see Table 3). Note: The initially lower success rate of FRMD during the first few thousand steps is due to the consistency distillation initialization from the teacher model, which requires adaptation to the single-step inference regime.

Figure 2 presents the learning curves throughout the training process for all 12 tasks. Several key observations emerge from this analysis.

**Convergence Behavior.** FRMD exhibits a characteristic two-phase learning pattern: during the initial 5,000 training steps, success rates are lower than the teacher MPD as the student network adapts to single-step generation. However, after approximately 10,000 steps, FRMD rapidly improves and surpasses both baselines. On hard tasks (e.g., PegInsertion-v1, PlugCharger-v1), FRMD converges faster than MPD, reaching peak performance around 20,000 steps compared to MPD’s 25,000 steps.

**Training Stability.** The shaded regions in Figure 2 represent variance across random seeds. FRMD consistently exhibits lower variance than DP across most tasks, indicating more stable learning dynamics. This stability likely results from the combination of (1) structured motion primitives that constrain the action space and (2) the stable training targets provided by the EMA target network in consistency distillation.

#### 4.2.4 Motion Quality Analysis

To assess trajectory smoothness, we evaluate both qualitative trajectory visualizations and a curvature-based smoothness metric. We focus on the PlugCharger-v1 task (medium difficulty) as a representative example and fix the initial environment state across trials to ensure comparable conditions.

**FIGURE 3:**
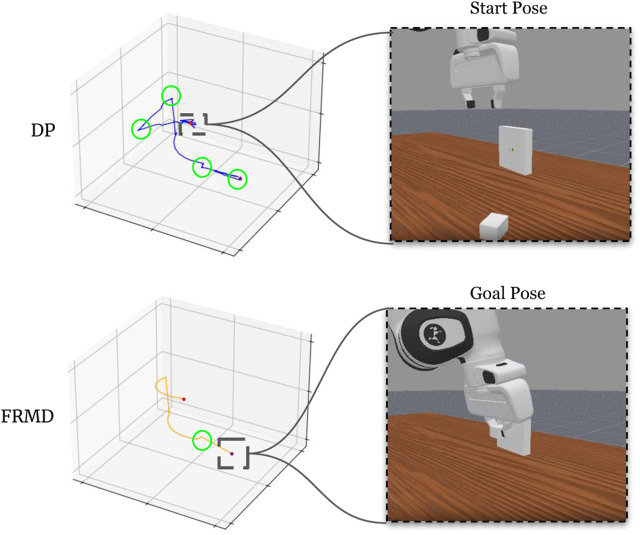
End-effector trajectory comparison on PlugCharger-v1 task. Top: trajectory generated by Diffusion Policy (DP). Bottom: trajectory generated by FRMD (ours). Green circles highlight regions where the trajectory curvature exceeds the smoothness threshold ( $\kappa_{i}>\kappa_{\max}=1.0$ rad/m), indicating abrupt directional changes. DP produces 82 non-smooth transitions with noticeable oscillations, particularly near the start and goal configurations. In contrast, FRMD generates a significantly smoother trajectory with only 21 non-smooth transitions, demonstrating superior motion stability and more natural, human-like motion characteristics.

**Quantitative Analysis.** Figure 3 shows the end-effector trajectories generated by each method. We quantify trajectory smoothness by counting the number of non-smooth transitions, defined as time steps where curvature exceeds a fixed threshold. Using this metric, Diffusion Policy (DP) exhibits $N_{\text{DP}}=82$ non-smooth transitions, while FRMD exhibits $N_{\text{FRMD}}=21$ . This corresponds to a 74% reduction in non-smooth transitions for FRMD under the same task and initial conditions.

**Implications for Control Stability.** From a control perspective, trajectories with fewer abrupt changes may reduce sensitivity to execution noise and lead to more predictable behavior during contact-rich manipulation. The reduction in non-smooth transitions observed for FRMD indicates that the generated motions are more temporally consistent, which is a desirable property for real-world robotic deployment.

### 4.3 Ablation study: Impact of network architecture

We study the effect of the policy backbone architecture on FRMD’s performance and inference latency. Three alternative architectures are evaluated while keeping all other components fixed, including the observation encoder, ProDMP decoder, and training configuration.

The following architectures are considered.

- **Transformer (Default):** A transformer-based architecture with 6 layers, 4 attention heads, and hidden dimension 256 (approximately 8.4M parameters). This configuration is used in all main experiments.
- **Transformer-Lite:** A reduced transformer variant with 3 layers, 2 attention heads, and hidden dimension 128 (approximately 2.1M parameters).
- **MLP:** A fully connected feedforward network with four hidden layers of sizes [512, 512, 256, 256] (approximately 3.2M parameters), without explicit temporal attention.

Table 4 reports success rates and inference latency across task difficulty levels.

**TABLE 4:** Ablation study on backbone network architectures.

Architecture	Easy (4)		Medium (5)		Hard (3)	
	Time	SR	Time	SR	Time	SR
Transformer	15.2	**99.2**	18.6	66.3	17.9	**29.0**
Transformer-Lite	16.2	98.5	15.8	**68.5**	15.9	26.2
MLP	**9.9**	74.9	**10.3**	54.2	**8.9**	9.2

We compare success rate (SR, %) and inference time (Time, ms) across different architectural choices. The best results in each column are highlighted in **bold**.

On easy tasks, all architectures achieve high success rates, with only minor differences between variants. The default transformer achieves a success rate of 99.2%, while Transformer-Lite and the MLP achieve comparable performance.

On medium-difficulty tasks, the Transformer-Lite variant attains the highest success rate (68.5%), slightly exceeding the default transformer (66.3%), while also reducing inference latency. This suggests that reduced-capacity transformer architectures can be sufficient for tasks of moderate complexity.

On hard tasks, performance differences between architectures become more pronounced. The default transformer achieves the highest success rate (29.0%), followed by Transformer-Lite (26.2%). In contrast, the MLP baseline exhibits a substantial drop in performance (9.2%), despite having the lowest inference latency.

Across all task categories, inference latency decreases with model capacity. The MLP achieves the lowest latency (8.9–10.3 ms), followed by Transformer-Lite (15.8–15.9 ms), and the default transformer (17.2–17.9 ms). However, this reduction in latency is accompanied by reduced task success on more complex tasks.

### 4.4 Limitations and future work

While FRMD achieves strong performance across a range of simulated manipulation tasks, this work has several limitations that suggest directions for future investigation.

**Applicability to Predictable Environments.** The proposed method is motivated by settings that require online action generation under uncertainty, such as contact-rich manipulation or partially observable environments. In tasks that are fully predictable or where accurate open-loop trajectories can be precomputed, the advantage of online diffusion-based generation may be reduced. In such cases, deterministic or preplanned controllers may achieve comparable performance without the overhead of generative sampling. The current work does not explicitly compare against open-loop trajectory optimization in fully predictable settings, and the relative benefit of FRMD in those scenarios remains to be further studied.

**Evaluation in Simulation.** As the evaluation is conducted in simulation, the reported latency and motion-quality results are limited to simulation conditions. Inference latency is measured on the target compute platform, but does not include delays introduced by sensing, communication, or low-level control loops in a physical robot. In real deployment, sensor noise, state-estimation errors, and actuation delays may affect both the achievable control frequency and the smoothness of the executed motion. Although the proposed method reduces the computational cost of action generation, its behavior under real-world disturbances may differ from what is observed in simulation. For instance, noisy observations could alter the predicted trajectory parameters, and additional execution delays may diminish the practical benefit of faster inference. Evaluating the method on physical hardware is left for future work.

**Scope of Motion Quality Analysis.** Our motion-quality analysis focuses on a representative medium-difficulty task (PlugCharger-v1), where we observe a substantial reduction in non-smooth transitions. Although results are consistent across multiple random seeds, we do not perform a comprehensive smoothness analysis across all tasks. Extending curvature-based metrics to the full task suite and incorporating statistical significance testing would provide a more complete characterization of trajectory quality.

**Trajectory Length and Task Structure.** The tasks evaluated in this work involve relatively short trajectories with fixed action horizons, which is consistent with common manipulation benchmarks. As a result, the current experiments do not assess how the proposed formulation behaves in longer-horizon settings or under variable-length execution. FRMD is designed for trajectory-level generation over a finite horizon, and extending it to handle longer, compositional, or dynamically re-planned trajectories would likely require additional mechanisms, such as hierarchical structure or horizon adaptation. We leave these extensions to future work.

**Offline Parameterization of Movement Primitives.** The movement-primitive parameterization used in this work, including the number and temporal placement of basis functions in the ProDMPs decoder, is specified offline following standard formulations. These parameters are chosen based on common practice and prior work, rather than being optimized for each task. As a result, we do not study how different parameter choices may affect expressiveness or motion quality, and a systematic exploration of this design space is left for future work.

**Integration with Vision-Language-Action Models.** Although FRMD is designed as a modular action-generation component, we do not evaluate it within a large-scale vision-language-action (VLA) model in this work. Investigating how FRMD interacts with pretrained vision-language encoders and whether its efficiency benefits persist in end-to-end VLA systems is left for future study.

## 5 Conclusion

We present Fast Robot Motion Diffusion (FRMD), a method that combines Consistency Models with Probabilistic Dynamic Movement Primitives (ProDMPs) (Paraschos et al., 2013) for efficient and smooth robotic motion generation. By distilling a Movement Primitive Diffusion (MPD) teacher model (Scheikl et al., 2024) through consistency training, FRMD achieves real-time, single-step inference while maintaining or exceeding the motion quality of multi-step diffusion baselines.

Our evaluation across 12 manipulation tasks from MetaWorld and ManiSkill demonstrates three key results: First, FRMD reduces non-smooth transitions by 74% compared to Diffusion Policy, generating more natural and stable trajectories. Second, FRMD achieves 10 × faster inference than MPD (17.2 ms vs. 168.6 ms) while maintaining superior task success rates (64.8% vs. 64.1%). Third, these improvements enable real-time robotic control at approximately 58 Hz, compared to 6–8 Hz for previous diffusion-based methods—a significant advancement toward practical deployment.

### 5.1 Broader impact and future directions

FRMD addresses a fundamental challenge in applying diffusion models to robotics: balancing generation quality with real-time computational requirements. The order-of-magnitude speedup from 168.6 ms to 17.2 ms per action sequence represents a practical breakthrough, making diffusion-based robot learning viable for responsive control applications.

Beyond the immediate results, several directions emerge for extending this work. Integration with large-scale Vision-Language-Action models represents a particularly exciting opportunity: FRMD’s modular action decoder could serve as an efficient “action expert” within VLA architectures, combining internet-scale vision-language pre-training with real-time motion generation.

More broadly, as robotic systems scale in complexity and capability, methods that effectively balance expressive power with computational efficiency will become increasingly critical. By demonstrating that structured motion representations and consistency distillation can achieve both high-quality generation and real-time performance, FRMD provides a blueprint for deploying powerful generative models in physically embodied systems where both quality and speed matter.

## Data Availability

The original contributions presented in the study are included in the article/supplementary material, further inquiries can be directed to the corresponding author.
